# Morphological, Rheological, and Mechanical Properties of Polyamide 6/Polypropylene Blends Compatibilized by Electron-Beam Irradiation in the Presence of a Reactive Agent

**DOI:** 10.3390/ma9050342

**Published:** 2016-05-06

**Authors:** Boo Young Shin, Man Ho Ha, Do Hung Han

**Affiliations:** 1 School of Chemical Engineering, Yeungnam University, Gyeongsan 712-749, Korea; dhhan@ynu.ac.kr; 2 R & D Center, Korea Petrochemical Limited, Ulsan 44785, Korea; hmh@kpic.co.kr

**Keywords:** compatibilization, PA6/PP blend, electron-beam irradiation, morphology, rheological properties, mechanical properties, glycidyl methacrylate (GMA)

## Abstract

An immiscible polyamide 6 (PA6)/polypropylene (PP) blend was compatibilized by electron-beam irradiation in the presence of reactive agent. Glycidyl methacrylate (GMA) was chosen as a reactive agent for interfacial cross-copolymerization between dispersed PP and continuous PA6 phases initiated by electron-beam irradiation. The PA6/PP (80/20) mixture containing GMA was prepared using a twin-screw extruder, and then exposed to an electron-beam at various doses at room temperature to produce compatibilized PA6/PP blends. The morphological, rheological, and mechanical properties of blends produced were investigated. Morphology analysis revealed that the diameter of PP particles dispersed in PA6 matrix was decreased with increased irradiation dose and interfacial adhesion increased due to high surface area of treated PP particles. Complex viscosities (η*) and storage moduli (G’) of blends increased with increasing irradiation dose and were higher than those of PA6 and PP. The complex viscosity of the blend irradiated at 200 kGy was 64 and 8 times higher than PA6 and PP, respectively. The elongation at break of blend irradiated less than 100 kGy was about twice that of PA6. Electron beam treatment improved the compatibility at the interface between PA6 and PP matrix in the presence of GMA.

## 1. Introduction

Polyamide 6 (PA6), commercially known as nylon 6, is a crystalline engineering thermoplastic which is tough, strong, and abrasion resistant and has a high melting temperature [1,2,3]. However, its high cost, low dimensional stability due water absorption, and low melt viscosity limits its utilization in specific applications [3]. For these reasons, PA6 can be blend with other thermoplastic as PP for improving desired properties, because PP is a high-volume cheap thermoplastic and shows lower water absorption, and higher melt viscosity.

Unfortunately, PA6 and PP are immiscible due to their structural and polarity differences [1,4], and, thus, several compatibilization strategies have been introduced [5,6,7]. Some research studies were conducted over recent years on the effects of irradiation on the properties of polymer blends [8,9,10], expecting cross-copolymerization (such as grafting or crosslinking) at the interface between the continuous and dispersed phases by using high-energy radiation without any reactive agent. However, to facilitate radiation-initiated cross-copolymerization effectively at the interface, a reactive agent is needed to be added [11,12]. Because electron-beam exposure process is usually performed at ambient temperature and immiscible blends have a gap at interface, which might be needed to fill before irradiation process. To the best of our knowledge, no previous study is reported describing the compatibilization of a PA6/PP blend by electron-beam-initiated interfacial cross-copolymerization in the presence of a reactive agent.

In this study, we compatibilized a PA6/PP blend using an electron-beam irradiation in the presence of reactive agent. Monomeric GMA was chosen as a reactive agent for interfacial cross-copolymerization because it has two reactive sites, namely, an epoxy functional group and a double bond. The epoxy group reacts easily with other functional groups during melt mixing [13,14,15] and the double bond can be easily opened by a radical species. Furthermore, due to low molecular weight, it can easily be diffused to the interface during melt mixing [16]. The morphological, rheological, and mechanical properties of the obtained blends were measured and analyzed to determine the effects of this proposed compatibilization strategy.

## 2. Results and Discussion

### 2.1. Morphology

**Figure 1:**
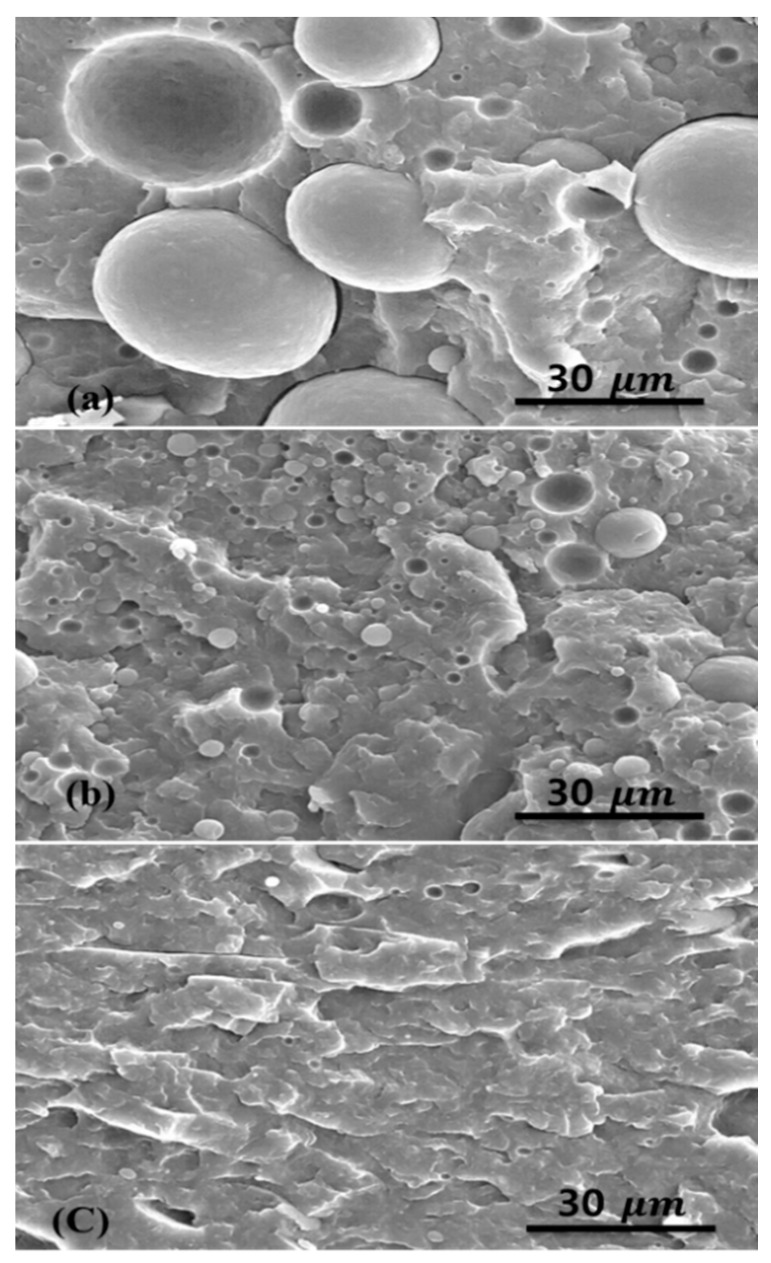
SEM images of cryofractured surfaces of PA6/PP (80/20) blends irradiated at 0 kGy (**a**); 20 kGy (**b**); and 200 kGy (**c**).

The cryofractured surface of the non-irradiated blend (Figure 1a) showed irregular and large imbedded PP particles with clear boundaries between the dispersed (PP) and continuous (PA6) phases. In Figure 1a, SEM images showed largest PP particles (diameters ≥30 μm) in non-irradiated sample. However, irradiation at 20 kGy markedly reduced the diameters of dispersed PP particles and weakened boundaries (Figure 1b). This trend continued further with high irradiation doses and finally these boundaries were not observed in the sample irradiated at 200 kGy (Figure 1c). This observed decrease in particle size is believed due to the reduction in interfacial tension between PA6 and PP components. Accordingly, we suggest that electron-beam irradiation in the presence of reactive agent GMA induced cross-copolymerization due to grafting and crosslinking at the interface between dispersed PP and PA6 continuous phases [10].

**Figure 2:**
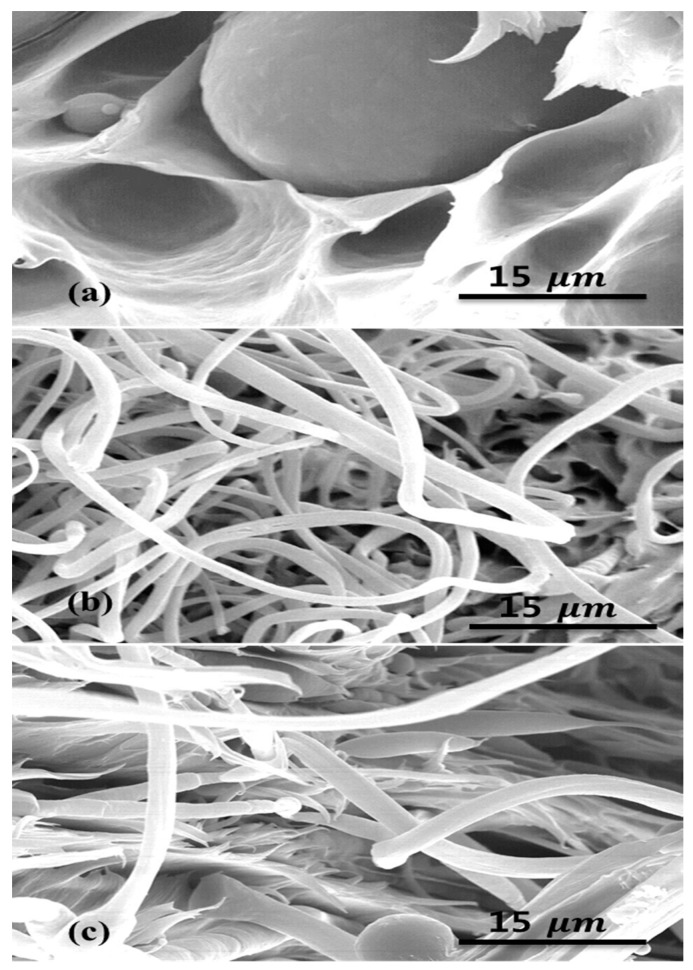
SEM images of the tensile fractured surfaces of PA6/PP (80/20) blends irradiated at 0 kGy (**a**); 20 kGy (**b**); and 200 kGy (**c**).

Tensile fractures can provide better insight of interfacial behaviors which, generally not observed on cryofracture surfaces. Therefore, we observed tensile fractured surface of blends irradiated at 0 kGy, 20 kGy, and 200 kGy, as shown in Figure 2. In non-irradiated sample, large spherical particles (diameter about 30 μm) were observed imbedded in elongated holes, which presumably were formed by pulled out PP particles. The spherical nature of these PP particles indicates a lack of interfacial adhesion and a failure to transfer tensile stress across the interface. On the other hand, sample irradiated at 20 kGy showed elongated PP microfibrils, indicating sufficient interfacial adhesion to transfer tensile stress. Sample produced at 200 kGy still showed microfibrils with greater diameter than that of sample irradiated 20 kGy due to reduced elongation of blend. These morphological results showed that the compatibility of PA6/PP blend was greatly enhanced, which presumably was due to interfacial cross-copolymerization induced by an electron-beam radiation process in the presence of reactive agent GMA.

### 2.2. Mechanisms of PA6/PP Compatibilization

**Scheme 1:**
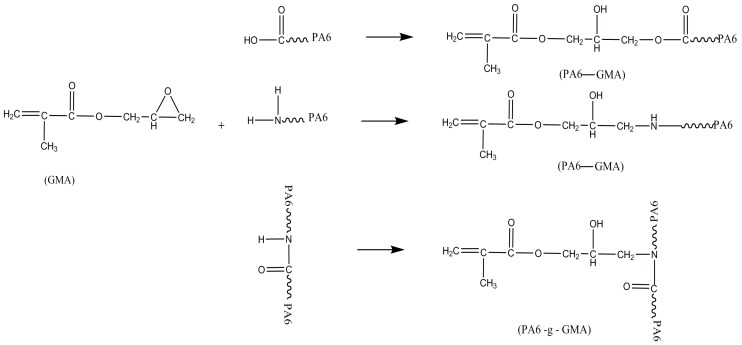
Schematics of the ring opening reaction occurring between the GMA and PA6 during melt mixing.

Here, we propose reaction mechanisms for the electron-beam-initiated cross-copolymerization with GMA as a reactive agent at the interface. Scheme 1 estimates ring opening (temperature dependent) reaction occurring between functional group of GMA and PA6 during the melt mixing, whereby the epoxy group of GMA reacts with primary amine and/or carboxylic acid end groups of PA6 to form linear PA6−GMA [14,17,18]. In addition, the epoxy group of GMA can react with the secondary amine of the amide to form GMA grafted PA6 (PA6−g−GMA) polymeric chains (branching).

**Scheme 2:**
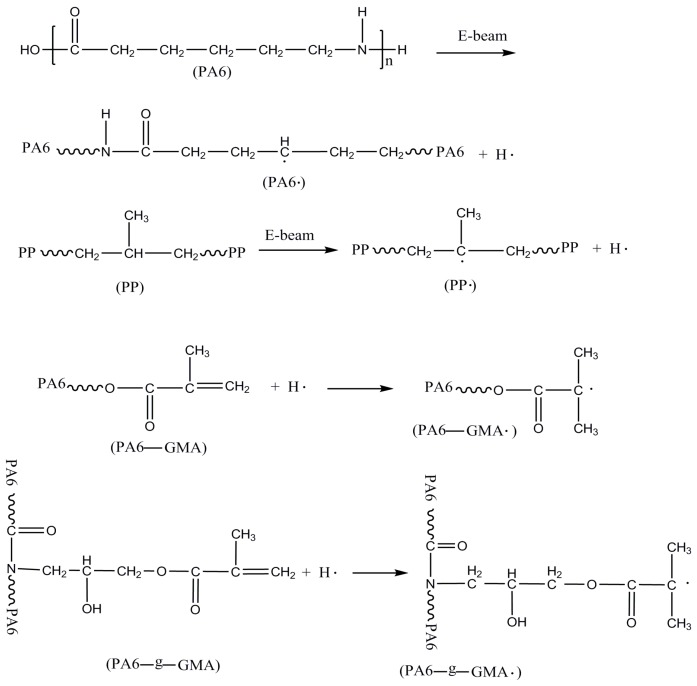
Electron-beam initiation schemes of the PA6, PP, PA6−GMA, and PA6−g−GMA.

Scheme 2 involved formation of macroradicals, namely PA6•, PP•, PA6−GMA•, and PA6−g−GMA•. The PA6• and PP• represent radicals formed by C−H cleavage at the arbitrary carbon atom sites of PA6 and hydrogen abstraction at the quaternary carbon atom sites of PP [19]. The PA6−GMA• and PA6−g−GMA• seems to be produced through opening the double bonds in the PA6−GMA and PA6−g−GMA by hydrogen radical (H•), as shown in Scheme 2.

**Scheme 3:**
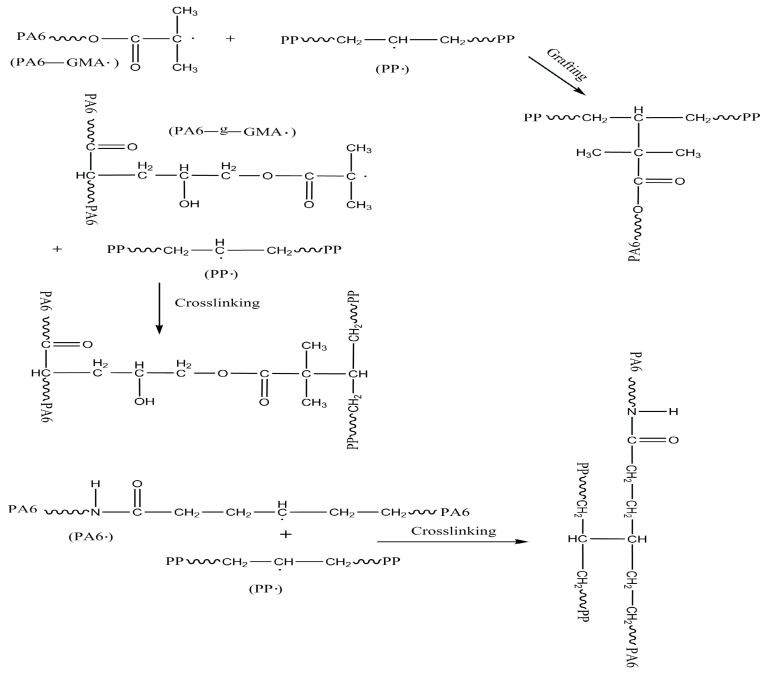
Suggested mechanisms of interfacial cross-copolymerization.

Scheme 3 estimates cross-copolymerization reactions (grafting and crosslinking) under active radical centers at the PA6−PP interface to produce hybrid branched macromolecules or network structures [20]. Grafting would involve PA6−GMA• and PP•, while crosslinking would involve PA6−g−GMA• and PP• and both are likely to increase interfacial adhesion between PA6 and PP phases. According to these schemes, the GMA seems to be very important agent for cross-copolymerization between PA6 and PP polymeric chains.

These cross-copolymerization reactions at interface would probably reduce the dispersed PP sizes by reducing the interfacial tension and stabilize the morphology by preventing the coagulation of dispersed particles, and greatly increase interfacial adhesion, as indicated by the morphological analysis of the treated samples [1].

### 2.3. Rheological Properties

**Figure 3:**
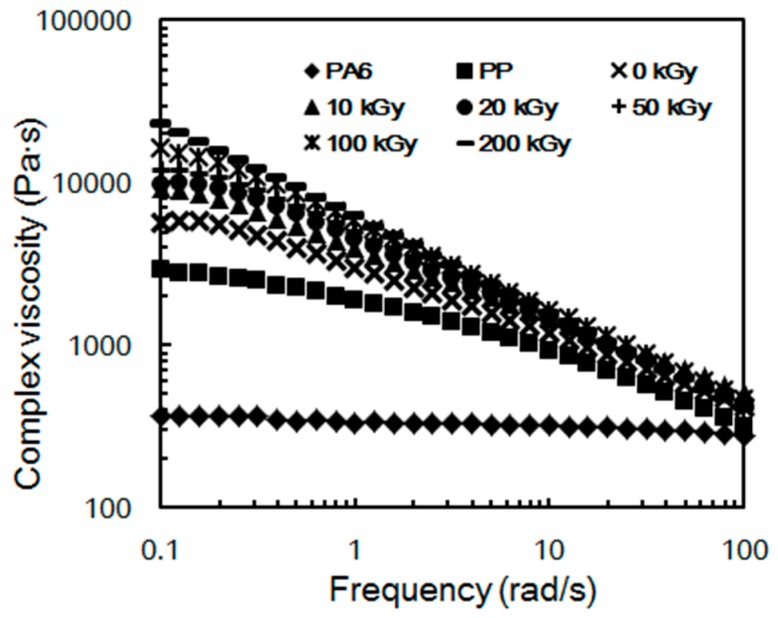
Complex viscosities of PA6/PP (80/20) blends irradiated at different dosages as a function of frequency at 235 °C.

The rheological properties of polymers are highly dependent on molecular weights and structures, such as, branching and crosslinking [21,22,23]. Figure 3 shows the complex viscosities of PA6, PP, and PA6/PP blends irradiated at 0 kGy, 10 kGy, 20 kGy, 50 kGy, 100 kGy, and 200 kGy. PA6 exhibited Newtonian flow behavior over the evaluated frequency range having a low melt viscosity of around 300 Pa∙s. On the other hand, PP had higher complex viscosities than PA6 and showed weak shear-thinning behavior. Remarkably, all PA6/PP blends had very higher complex viscosities than PA6 or PP and showed a significant shear-thinning behavior at all observed frequencies. At frequency of 0.1 rad/s, PA6/PP blends had 15~64 and 2~8 times higher complex viscosities than PA6 and PP, respectively. In addition, the dependency of complex viscosity on the frequency increased. The increase in the complex viscosity and non-Newtonian behavior of PA6/PP blends on increasing irradiation dose suggested increased interfacial adhesion.

**Figure 4:**
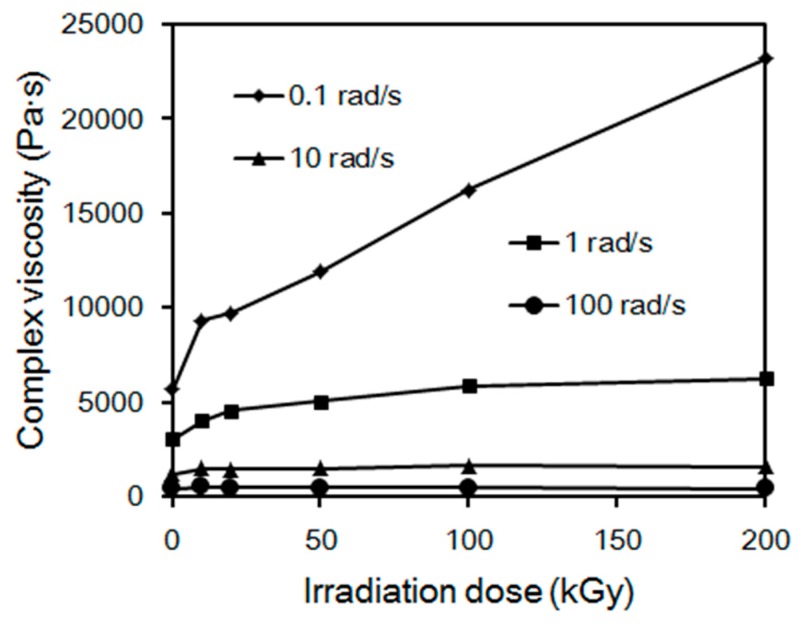
Complex viscosities of PA6/PP (80/20) blends as a function of irradiation dose at different frequencies.

Figure 4 shows a plot of complex viscosity versus irradiation dose at frequencies of 0.1 rad/s, 1 rad/s, 10 rad/s, and 100 rad/s. As shown in Figure 4, complex viscosity was observed to increase markedly at 0.1 rad/s on increasing irradiation dose, but complex viscosities at 1 rad/s, 10 rad/s, and 100 rad/s increased at lower rate on increasing irradiation dose. According to the literature [24], rheological properties at lower frequencies represent interfacial properties of polymer blends due to relatively long relaxation time of droplet shape. The above results indicate that compatibility of blend was improved by electron-beam irradiation in the presence of GMA and that compatibility was dependent on irradiation dose.

**Figure 5:**
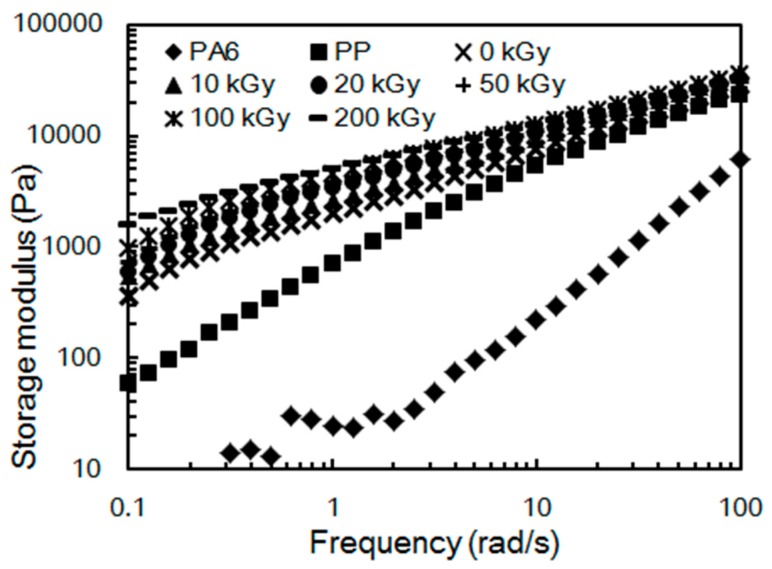
Storage moduli of PA6/PP (80/20) blends irradiated at different dosages as a function of frequency at 235 °C.

Figure 5 shows changes in the storage moduli of PA6, PP, and of PA6/PP blends irradiated at different doses. As shown by Figure 5, the storage moduli of all blends were higher than those of PA6 and PP over the entire frequency range [24,25]. Furthermore, storage moduli of PA6/PP blends increased with increasing irradiation dose and the storage modulus curves of blends converged at the same plateau modulus at higher frequencies. The storage moduli of pure PA6 are very low at a lower frequency range, resulting in fluctuation due to the detecting limit of ARES.

**Figure 6:**
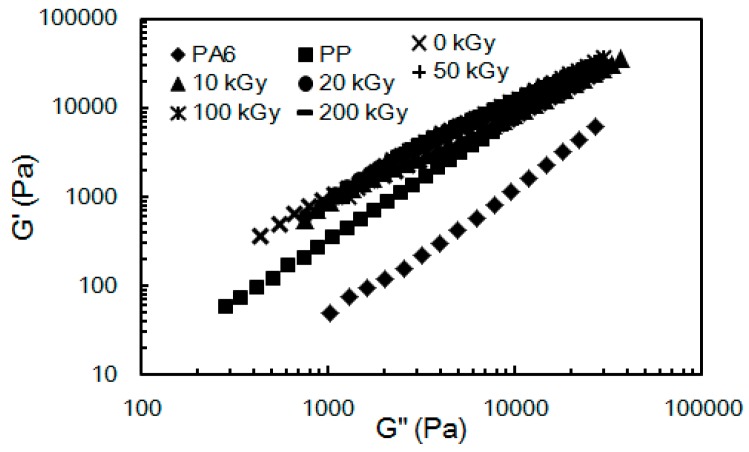
Plots of storage modulus (G’) against loss modulus (G”) for virgin PA6, PP, and PA6/PP (80/20) blends irradiated at different dosages.

Modified Cole-Cole plots, log storage modulus (G′) *vs.* log loss modulus (G″), are useful for analyzing structural change of polymer molecule, such as branching and crosslinking [21,22,23]. The effects of molecular weight, molecular weight distribution, branching, and crosslinking on modified Cole-Cole plot for various polymers have been investigated both experimentally and theoretically. In general, for linear polymers, the plot is not dependent on molecular weight but dependent on chain branching and crosslinking and slightly dependent on molecular weight distribution. As shown in Figure 6, the plots of blends were not coincident, presumably due to structural difference. 

These rheological results supported the morphological analysis results and suggested reactions. In addition, the dependence of rheological properties on irradiation dose presumably suggests that the compatibility of PA6/PP blends depends on irradiation dose. 

However, these rheological results could be affected by gel formation of constituent. Therefore, we extracted blend by dissolving PA6 in formic acid. Then, the main component of residue must be PP and the minor component might be gel of PA6 and PA6-*co*-PP induced by electron-beam irradiation in the presence of GMA. Unfortunately, it was difficult to separate gel of PA6 and PA6-*co*-PP from main component of PP. As listed in Table 1, there was no significant increase of residue with increasing irradiation dose, which indicates that the gel content of PA6 is negligible. In previous studies, neat PA6 irradiated from 40~150 kGy had 0% gel content [3] and PA6 irradiated at 200 kGy in the presence of GMA showed only 1.3% of gel content [18]. Accordingly, the increase in complex viscosity and modulus observed on increasing irradiation dose was not due to PA6 crosslinking by electron-beam irradiation but rather due to the cross-copolymerization at the interface, as shown in Scheme 3.

### 2.4. Mechanical Properties

**Figure 7:**
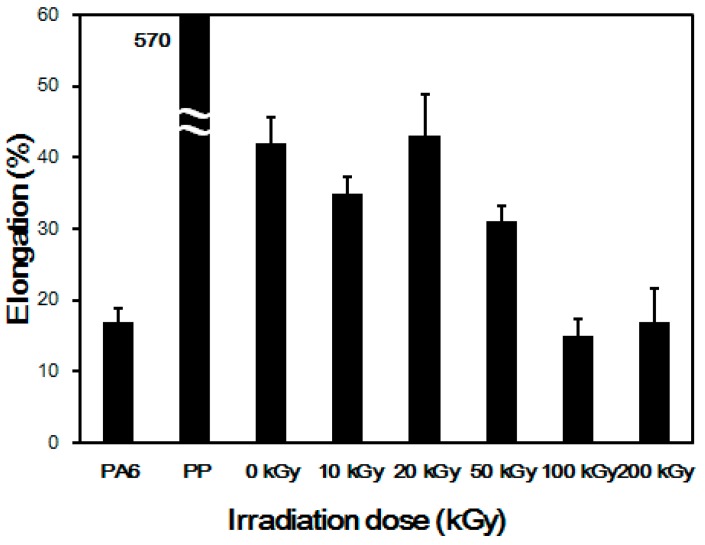
Effect of irradiation dose on the elongation at break of PA6/PP (80/20) blends.

**Figure 8:**
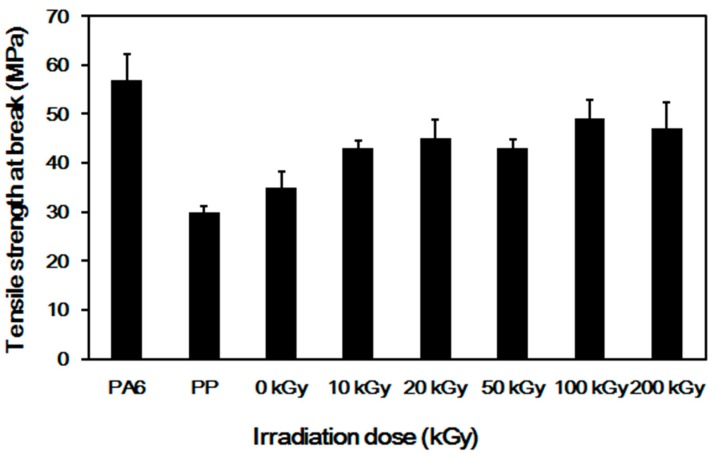
Effect of irradiation dose on the tensile strength at break of PA6/PP (80/20) blends.

Mechanical performance is an essential parameter for practical applications in the plastic industry and is dependent on improved compatibility of polymer blends. The changes in elongation at break of PA6/PP blends irradiated at different doses are shown in Figure 7. PA6/PP blends irradiated at less than 100 kGy, including non-irradiated blends, elongation at break values of PA6/PP blends were observed twice than that of pure PA6. In contrast, elongation at break values of PA6/PP blends irradiated 100 and 200 kGy were reduced to that of pure PA6; in spite of markedly increased compatibility as evidenced in the results of morphological investigation. The tensile strengths at break PA6/PP blends approximately increased with increasing irradiation dose due to increased compatibility, as shown in Figure 8. Mechanical properties might be also affected by crystallinity and gel content of constituents in blend. As shown in Table 1, melting temperature and crystallinity of PA6 were affected very little by irradiation dose; however, the crystallinity of PP increased up to 20 kGy and then decreased, which might be due to both the degradation and crosslinking of molecule [11]. The melting temperature of PP in the blend decreased with increasing irradiation dose from 164 °C (pure PP) to 151 °C (200 kGy) due to degradation. In addition, change in gel content of PA6 with irradiation dose was already discussed above and, therefore, the increase in elongation of blends compared with PA6 might be due to increased compatibility of blend. However, the decrease in elongation of blends irradiated over 100 kGy may be caused by degradation of PP, not by formation crosslinking or change in crystallinity of PA6 [11].

## 3. Experimental

### 3.1. Materials

Polyamide 6 (Domamid^®^ 24) with a density of 1.14 g/cm^3^ was obtained from DOMO Caproleum GmbH (Premnitz, Germany). Glycidyl methacrylate (GMA) and formic acid were provided by Sigma-Aldrich (Milwaukee, WI, USA). Polypropylene (YUHWA POLYPRO^®^ 4017M) with a density of 0.9 g/cm^3^ and melt flow index (MFI) of 14 g/(10 min) at 230 °C and 2.16 kg was obtained from Korea Petrochemical Inc. Co. Ltd. (Ulsan, Korea).

### 3.2. Melt Mixing of PA6, PP, and GMA

The blend ratio 80/20 (weight percent) PA6 to PP was chosen and the amount of GMA was fixed at 3 parts per hundred resin (phr) based on the total mass of PA6 and PP. PA6, PP, and GMA were mixed in a plastic bag before being extruded in a twin-screw co-rotating extruder (SM PLATEK Co. Ltd., TEK 30MHS, Ansan, Korea). Screw diameter was 31.6 mm with 40:1 L/D ratio. The extruder was operated at 200 rpm with a constant feed rate of 20 kg/h. The barrel and die temperatures were set at 200~240 °C and 245 °C, respectively. The extrudate was immediately cooled in chilled water and cut into pellets of a diameter less than 1 mm. Then, the pellets were dried for 24 h at 80 °C prior to the electron-beam irradiation.

### 3.3. Electron-Beam Irradiation

Obtained pellets were irradiated using a commercial electron-beam accelerator (ELV-0.5, BINP, Novosibirsk, Russia) with a maximum beam current of 40 mA and a beam energy range of 0.5–0.7 MeV under a nitrogen atmosphere at room temperature. The irradiation doses were 10 kGy, 20 kGy, 50 kGy, 100 kGy and 200 kGy, which were controlled by varying beam current from 0.5 to 10 mA and a conveyor speed from 1 to 2 m/min. The irradiation doses were measured using film dosimeters (B3 WINdose Dosimetry, GEX Co., Centennial, CO, USA) and a dosimeter (GENESYS 20, Thermo SCIENTIFIC Co., Waltham, MA, USA). Acceleration voltage was 0.7 MeV and the effective penetration depth was about 2 mm for a substrate of density 1 g/cm^3^ [26,27]. The irradiated samples were dried in an oven at 80 °C for 12 h to eliminate residual radicals.

### 3.4. Characterization

The morphologies of the blends were examined by observing cryogenic and tensile fracture surfaces using a scanning electron microscope (SEM, Hitachi model s-4200, Tokyo, Japan). Rheological properties were measured using an ARES (Advanced Rheometric Expansion System: Rheometric Scientific Co. Ltd., New Castle, DE, USA) rotational rheometer. The equipment was run in parallel plate configuration at 235 °C and a strain of 2% in the angular frequency range of 0.1 to 100 rad/s. Mechanical properties of the blends were determined using INSTRON 4464 tensile tester (INSTRON, Norwood, MA, USA). Tests were performed on tensile bars (type II) that were compression molded according to the KS M3600 test method using a hot press (Model 3851-O, Carver Inc., Wabash, IN, USA) at a set temperature of 240 °C and a molding pressure of 14 MPa. The experiment was performed at room temperature with a gauge speed of 10 mm/min and a gauge length of 35 mm. All tests were performed in four fold and only the average value was reported. Thermal properties were determined using differential scanning calorimetry (DSC; TA INSTRUMENTS Q200, New Castle, DE, USA). Samples were heated from room temperature to 250 °C at a rate of 20 °C/min and maintained at 250 °C for 3 min to remove the thermal history. Subsequently, they were quenched to −30 °C then reheated to 250 °C at 10 °C/min under a nitrogen atmosphere. The degree of crystallinity, X_c_, was calculated as:

(1) $$X_{c}(\%)=\frac{{∆H}_{f,i}/\phi_{i}}{{∆H}_{f,i}^{o}}\times 100$$

where ${∆H}_{f,i}$ and $∆H_{f,i}^{o}$ are the enthalpies (J/g) of fusion of the blend and 100% crystal components, respectively. $\phi_{i}$ is the mass fraction of component in the blend. $∆H_{f}^{o}$ of PA6 and PP are 188.1 J/g [3] and 209 J/g [28], respectively. The prepared PA6/PP blends irradiated at the doses of 0 kGy, 10 kGy, 20 kGy, 50 kGy, 100 kGy and 200 kGy were separated by extraction in formic acid for 48 h using Soxhlet extractor. The formic acid is a good solvent for PA6 but a non-solvent for PP. The % of residue of extracted blend was calculated as follows:

*Residue (%) = W_r_/W_o_ × 100,* (2)

where *W_r_* is the weight of residue after extraction with boiling formic acid *W_o_* is the weight of specimen before extraction.

## 4. Conclusions

In summary we studied the effect of electron-beam exposure in the presence of reactive agent on the compatibility of PA6/PP (80/20) blend, in which interfacial cross-copolymerization was induced. Microscopic studies of cryofractured and tensile fractured sample morphologies revealed that dispersed particle size of PP decreased and interfacial adhesion increased on increasing irradiation dose. At a frequency of 0.1 rad/s, complex viscosities and storage moduli of all PA6/PP blends were 15~64 and 2~8 times greater than those of pure PA6 and PP, respectively. In addition, elongation at break values of PA6/PP blends were observed about twice than that of PA6 when irradiated at 0 kGy, 10 kGy, 20 kGy and 50 kGy. The observed morphological improvement and enhanced rheological and mechanical properties of irradiated PA6/PP blends supported the formation of PA6-*co*-PP copolymer at the interface, wherein GMA acted as a reactive agent. Furthermore, these findings show that the compatibility of PA6/PP (80/20) blend containing a reactive agent can be improved by exposure to electron-beam irradiation.

## Figures and Tables

**Table 1:** Thermal properties of PA6, PP, and blends, and extraction result of blends.

Sample	*T*_*m*1_ (°C)	*X_c_* (%) of PA6 in blend	*T*_*m*2_ (℃)	*X_c_* (%) of PA6 in Blend	Residue (%)
PA6	–	–	216	32.0	–
PP	164	33.1	–	–	–
Blend irradiated at 0 kGy	157	31.7	216	28.5	20.1
Blend irradiated at 10 kGy	156	39.5	216	28.2	20.5
Blend irradiated at 20 kGy	155	39.4	216	24.6	20.8
Blend irradiated at 50 kGy	155	26.3	217	27.8	20.3
Blend irradiated at 100 kGy	153	26.4	215	27.1	20.5
Blend irradiated at 200 kGy	151	27.0	214	27.5	21.8

*T*_*m*1_ and *T*_*m*2_ are the melting temperatures of PP and PA6 phases in the blend respectively.
